# Epidemiological and clinical trends of visceral leishmaniasis in Portugal: retrospective analysis of cases diagnosed in public hospitals between 2010 and 2020

**DOI:** 10.1186/s40249-024-01204-5

**Published:** 2024-06-01

**Authors:** Rafael Rocha, Cláudia Conceição, Luzia Gonçalves, LeishPT group, Ana Cláudia Carvalho, André Maia, André Martins, António Carujo, António Maio, Catarina Forra, Catarina Melita, Daniela Couto, Diana Fernandes, Dulce Pereira, Ema Leal, Helena Sarmento, Inês Sousa, Jean-Pierre Gonçalves, Joana Marinho, Joana Vasconcelos, João Cunha, João Rodrigues, José Miguel Silva, Lídia Caley, Luís Malheiro, Luís Santos, Margarida Garcia, Margarida Prata, Maria Cunha, Maria Lima, Maria Margarida Andrade, Marta Marques, Miguel Alpalhão, Mónica Silva, Rita Ferraz, Rui Soares, Salomão Fernandes, Samuel Llobet, Sofia Cruz, Teresa Guimarães, Tiago Branco, Tomás Robalo-Nunes, Vasco Almeida, Carla Maia

**Affiliations:** 1 Institute of Hygiene and Tropical Medicine (IHMT), University Novaof Lisbon (UNL), Rua da Junqueira Nº100, Lisboa, 1349-008 Portugal; 2 Global Health and Tropical Medicine (GHTM), Associate Laboratory in Translation and Innovation Towards Global Health, LA- REAL, IHMT, UNL, Rua da Junqueira Nº100, Lisboa, 1349-008 Portugal; 3 University Hospital Center of São João, Alameda Prof. Hernâni Monteiro, Porto, 4200-319 Portugal; 4 Faculty of Sciences, Centre of Statistics and its Application of the University of Lisbon, University of Lisbon, Campo Grande, Lisboa, 1749-016 Portugal; 5 Z-Stat4life, Cowork Space Baldaya, Baldaya Palace, Estrada de Benfica Nº 701ª, Lisboa, 1549-011 Portugal

**Keywords:** *Leishmania*, Leishmaniasis, Visceral, People living with HIV, Children, Portugal

## Abstract

**Background:**

*Leishmania infantum* is endemic in the Mediterranean region, presenting mostly as visceral leishmaniasis (VL). In Portugal, reporting of VL cases to public health authorities is mandatory, but significant underreporting is likely. This study aimed to describe the epidemiological and clinical aspects of the VL cases diagnosed in hospitals of the Portuguese National Health Service (NHS), between 2010 and 2020.

**Methods:**

Collaboration was requested to every hospital of the Portuguese NHS in Mainland Portugal. Cases were screened through a search of diagnostic discharge codes or, if not available, by a search of positive laboratory results for *Leishmania* infection. Sociodemographic and clinical data was retrieved from medical records. Simultaneously, the National Health authority was contacted to request access to data of notified cases of VL between 2010 and 2020. Descriptive, hypothesis testing and multiple binary logistic regression models were performed.

**Results:**

A total of 221 VL cases were identified. A significant increase in estimated national incidence was seen in the years after 2016 (*P* = 0.030). VL was predominantly diagnosed in people living with HIV (PLWH) and in children (representing around 60% of the new cases), but the outcome was generally poorer in non-HIV patients with associated immunosuppression, with significantly lower rates of clinical improvement at 7 (*P* = 0.003) and 30 days (*P* = 0.008) after treatment. Atypical presentations, with gastrointestinal and/or respiratory involvement, were seen in 8.5% of VL cases. Hemophagocytic lymphohistiocytosis was diagnosed in 40.0% of children under 5 years of age. Only 49.7% of incident VL cases were reported. Simultaneous involvement of the skin was confirmed in 5.9% of patients.

**Conclusions:**

VL presents a continuing threat in Portugal, especially to PLWH and children, and an increasing threat to other immunosuppressed groups. Recent increases in incidence should be closely monitored to allow prompt interventions. Programs to control the disease should focus on providing tools for earlier diagnosis and on reducing underreporting and promoting an integrated surveillance of human and animal disease. These data should be combined with asymptomatic infection and vector information, following a One Health approach.

**Graphical Abstract:**

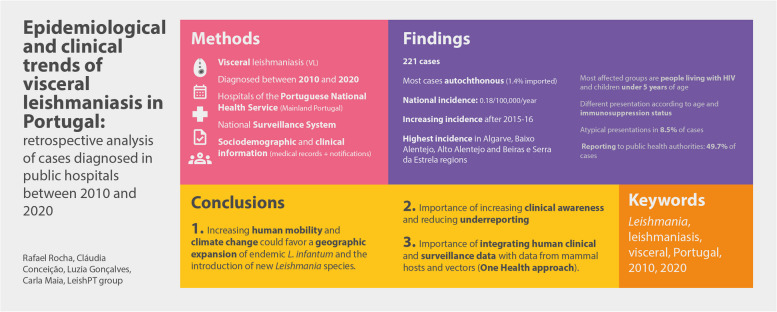

**Supplementary Information:**

The online version contains supplementary material available at 10.1186/s40249-024-01204-5.

## Background

Leishmaniases are a group of diseases caused by protozoan parasites of the genus *Leishmania*. These parasites are transmitted by phlebotomine sand flies, and the disease is zoonotic in most settings [1]. Clinical spectrum of symptomatic disease is usually grouped into two main syndromes, visceral leishmaniasis (VL) and cutaneous leishmaniasis (CL) [1], both of which are endemic and geographically widespread in the Mediterranean region. In this region, *L. infantum*, which belongs to the *L. donovani* complex, is the etiologic species of most autochthonous human leishmaniasis cases [2]. Infection with *L. infantum*, when symptomatic, usually presents as VL, although cases of simultaneous or independent CL and mucosal leishmaniasis caused by this species are increasingly recognized [3].

In the western Mediterranean regions where *L. infantum* is endemic, including in Portugal, *Phlebotomus perniciosus* is the main vector [4], and dogs are considered to be the main reservoir for human infection [5]. Increasing evidence suggests that cats [6] and some wild animals (such as leporids [7]) may also play a relevant epidemiological role.

An important share of symptomatic *L. infantum* infection in Southern Europe has been described in people living with human immunodeficiency virus (HIV) and children [8]. However, cases in the context of non-HIV related immunosuppression have been recently increasingly described, including solid organ transplant recipients and patients with autoimmune and inflammatory diseases chronically medicated with immunosuppressive drugs [9].

In the period from 2005 to 2020, 5813 VL cases were reported to the WHO in the European region [10]. The cumulative incidence in this period per 100,000 population of VL was highest in Albania (2.15 cases), followed by Montenegro, Malta, Greece, Spain and North Macedonia (0.53–0.42), Italy (0.16), Portugal (0.09). However, for several countries, incidence estimates according to hospital discharges were significantly higher than calculated using WHO reported cases [10].

In Portugal, reporting of VL cases to central public health authorities is mandatory, as part of a passive surveillance system. The most recent findings from this system showed that 6 to 14 cases were reported per year between 2014 and 2018 [11], although this likely represents a significant underreporting of cases, as revealed in a previous study where, between 1999 and 2009, only 38.6% of cases diagnosed in public hospitals were notified to central public health authorities [12].

This study aimed to describe the epidemiological and clinical aspects of the cases of VL diagnosed in hospitals of the Portuguese National Health Service, between 2010 and 2020, as well as those reported to public health authorities over the same period.

## Methods

### Study population

This multicenter retrospective study targeted all cases of leishmaniasis diagnosed in public hospitals in Mainland Portugal, between 2010 and 2020. Mainland Portugal is located in Southwest Europe, bordering Spain and the Atlantic Ocean. According to the 2021 national census, the population of mainland Portugal was 9,857,593 inhabitants [13], of which 542,165 (5.2%) were born abroad [14]. Mainland Portugal is divided into five NUTS2 (from the French *Nomenclature des Unités Territoriales Statistiques*, Nomenclature of Territorial Units for Statistics) regions, 23 NUTS3 regions (Supplementary Fig. 1 and Supplementary Table 1), 278 municipalities and 2882 parishes. Between 2010 and 2020, hospital-based healthcare services were provided by the Portuguese National Health Service in 102 to 111 general and specialized hospitals in Mainland Portugal, according to data from the Directorate-General for Health (DGS) of Portugal [15]. Some of these hospitals are grouped in Hospital Centers. Every episode of admission to these hospitals as an emergency or inpatient is given a code on discharge for primary and secondary diagnoses, following the International Classification of Diseases (ICD). Mandatory notifications of VL cases to central health authorities, initially done in paper format, have, since 2014, been submitted through an electronic platform, the National Epidemiologic Surveillance System (SINAVE) [16].

Individuals diagnosed with VL in one of the hospitals of the Portuguese National Health Service, in Mainland Portugal, were included in this study. No age restrictions were considered, and both inward and outpatient settings were accepted. Only laboratory confirmed cases were included. This consisted of the presence of a compatible clinical picture and meeting at least one of the following criteria: (i) Detection of antibodies against *Leishmania* in serum; (ii) Detection of *Leishmania* DNA in any biological sample; (iii) Visualization of intracellular organisms in macrophages, compatible with *Leishmania* amastigotes in biopsy material or cytological examination; (iv) Growth of *Leishmania* from any clinical sample inoculated in a specific culture medium.

### Data collection

Every hospital or hospital center was contacted and collaboration in this study was requested. Cases in each included hospital were screened through a search of diagnostic discharge codes: 085, 085.0, 085.9 (ICD-9); B55, B55.0, B55.9 (ICD-10). In hospitals where codification of diagnosis was incomplete or unavailable for the whole or parts of the period of analysis, listing of cases was complemented by searching positive *Leishmania* serology results and positive *Leishmania* DNA detection by Polymerase Chain Reaction (PCR) in the database of the Pathology laboratory. Additionally, cytology and histopathology reports (all types of samples) were screened for the keyword “*Leishmania*”. Reports where the word was identified were thoroughly read and selected for analysis if they mentioned observation of *Leishmania* amastigotes. Sociodemographic and clinical data of the cases identified (including clinical presentation, underlying conditions/comorbidities, diagnosis, management, and outcome) was extracted from the medical records of each episode, codified, and inserted into a digital database. Data extraction was carried out by different professionals; a common database was used and a protocol for filling in the required information was provided to every collaborator.

Simultaneously, the DGS was contacted and access to notified cases of VL between 2010 and 2020 was requested. Sociodemographic and clinical data of these cases was provided by the DGS in a codified database. Cases of VL obtained from the two sources (hospitals and notifications) were matched, considering the following individual details: age and sex of patient, municipality of residence at the time of diagnosis, date of notification or admission to hospital. For duplicated cases, data from both sources was merged into a single entry in the final database.

Categorical variables extracted from the clinical records or notifications were analyzed mostly using the original categories provided as options in the standardized database, but regrouping was performed in some cases. Non-improvement was defined as persistence or worsening of signs/symptoms or laboratory changes, despite appropriate therapy, and was assessed at seven and thirty days after starting treatment. These two timeframes were defined by the authors to allow homogeneous data collection regarding outcome in the different hospitals involved. Clinical improvement in VL (with resolution of fever) is usually evident at seven days, according to previous knowledge [1]. In addition, European guidelines propose a definition of non-response for VL as no clinical improvement at four weeks after start of therapy [17]. Relapses were defined as recurrence of signs/symptoms and positive culture/PCR/microscopy in blood or other biological sample after completing primary treatment with clinical improvement at 30 days. Other definitions, classifications or categories used for data collection and presentation in this study are presented in Supplementary Table 2.

### Statistical analysis

Annual mean incidence of VL was estimated based on the following formula: Incidence = (New Cases) / (Population × Timeframe), considering a timeframe of 11 years and an at-risk population, for each region, consisting of the average value between the number of inhabitants estimated in the census of 2011 and the census of 2021, according to the National Institute of Statistics [13]. The corresponding 95% confidence intervals (CIs) for the incidence rate were obtained using a substitution method [18].

Descriptive statistics and hypothesis testing were performed using IBM® SPSS® Statistics (Version 29.0, IBM Corp, Armonk, United States of America - USA). Bar charts were built using Microsoft® Excel® (Version Office 365, Microsoft Corp, Redmond, USA). Geographical representation and analysis of results was obtained using QGIS® (Version 3.22, Open Source Geospatial Foundation, Beaverton, USA).

For categorical variables, absolute frequencies and percentages were calculated. Symmetric continuous variables were summarized by means with standard deviations and asymmetric continuous variables (e.g., age, analytical values) by medians with interquartile intervals (IQIs). Missing or unknown data were excluded from denominators, unless stated otherwise.

For analysis of clinical variables, VL patients were distributed in four groups: children 5 years old or younger; non-immunosuppressed individuals over 5 years old; people living with HIV (PLWH); and non-HIV infected immunosuppressed individuals. Comparisons between these groups were performed using Pearson Chi-Square test (CST) for categorical variables; or Fisher’s exact test (FET) in case of failure of the assumptions of the CST. For continuous variables, after checking the assumptions of normality and homogeneity of the variances, the Mann-Whitney U test (MWT) or the Kruskal-Wallis test (KWT) were used, for comparing two or more independent groups, respectively. To compare survival distributions between two or more groups, the logrank test was used. A value of *P* < 0.05 was considered statistically significant.

To identify sociodemographic and clinical factors associated with non-improvement at 7 days after starting treatment and non-reporting of VL cases, multiple binary logistic regression models were explored, analyzing variables with statistical meaning in the univariate analysis (*P* < 0.20) and some biologically relevant or potentially confounding variables. For those variables that remained significant, crude odds ratio (*OR*) were updated to adjusted odds ratio (a*OR*) with 95% *CI*. The Hosmer–Lemeshow test was used for assessing goodness of fit in each multiple logistic regression model [19]. The reference categories used for each independent variable are specified in each results table.

## Results

### Sociodemographic characteristics and comorbidities

Data from 42 of the 45 hospitals or hospital centers in Mainland Portugal was available for analysis.

Sociodemographic characteristics of VL cases are represented in Table 1. A total of 221 cases of VL were diagnosed between 2010 and 2020 in the hospitals included: 201 as primary (or incident) cases and 20 as relapsing cases (first episode diagnosed before 2010). Of the 114 cases provided by the DGS, notified during this period, all but 13 were also identified through the hospital searches.

**Table 1:** Sociodemographic characteristics of visceral leishmaniasis cases diagnosed in public hospitals in Mainland Portugal in 2010–2020

Number	221
**Median age, years (IQI)**	41
	[28–50]
**Male sex, % (** ***n*** **)**	74.2
	(164/221)
**Region of diagnosis (NUTS2), % (** ***n*** **)**	
Norte	17.2
	(38/221)
Centro	15.8
	(35/221)
Área Metropolitana de Lisboa	49.8
	(110/221)
Alentejo	5.9
	(13/221)
Algarve	11.3
	(25/221)
**Country of birth, % (** ***n*** **)**	
Native	80.9
	(157/194)
Migrant^a^	19.1
	(37/194)
**Origin of infection, % (** ***n*** **)**	
Autochthonous	98.6
	(214/217)
Imported	1.4
	(3/217)^b^
**Travel/residence abroad in the previous 12 months, % (** ***n*** **)**	
Yes	8.9
	(9/101)
**Occupation, % (** ***n*** **)**	
Unemployed	24.2
	(23/95)
Retired	17.9
	(17/95)
Service and sales, craft and industry	29.5
	(28/95)
Agriculture and elementary	17.9
	(17/95)
Professionals, technicians and clerical support	8.4
	(8/95)
**Type of home, % (** ***n*** **)**	
Detached house	58.3
	(35/60)
Apartment	18.3
	(11/60)
Other^c^	23.3
	(14/60)
**Regular contact with domestic animals, % (** ***n*** **)**	
Yes	73.4
	(58/79)
Dogs	98.0
	(50/51)
Cats	13.7
	(7/51)
Other(s)^d^	17.6
	(9/51)

* IQI* Interquartile interval

^a^Angola *n*  = 9, Cape Verde *n*  = 6, Guinea-Bissau *n*  = 5, Brazil *n*  = 4, São Tomé e Príncipe *n*  = 3, Mozambique *n*  = 2, Senegal *n*  = 1, Eritrea *n*  = 1, Sweden *n*  = 1, Ukraine *n*  = 1

^b^Brazil *n* = 2, East Africa *n* = 1

^c^Homeless *n*  = 7, Shelter or nursing home *n*  = 6, Prison *n*  = 1

^d^Cattle/sheep/goat *n*  = 3, birds *n*  = 7, rabbit *n*  = 2

**Fig. 1:**
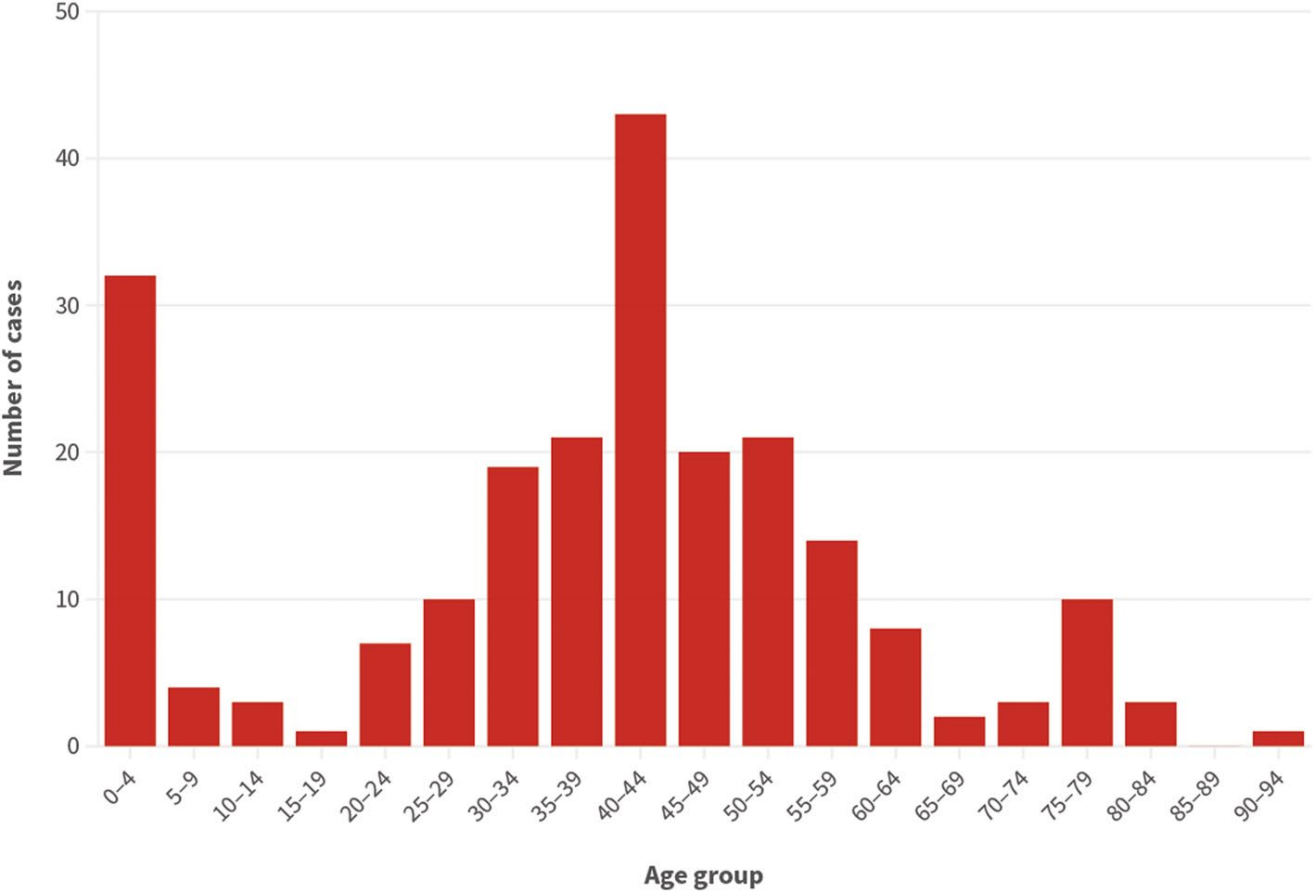
Age distribution (in years) of cases of visceral leishmaniasis diagnosed in 2010–2020 (*n* = 221)

Median age was 41 years old (IQI: 28–50) and male sex was predominant. Age distribution of cases of VL is represented in Fig. 1. Approximately half of the cases were diagnosed in hospitals in the Lisbon Metropolitan Area (Área Metropolitana de Lisboa - AML) region. Only three cases (1.4%) were imported (from Brazil *n* = 2 and East Africa *n* = 1). Migrants represented approximately 20% of patients diagnosed, most of them born in sub-Saharan Africa (27/33) or Brazil (4/33). The two most common occupation status reported, accounting for around half of all patients, were unemployment (24.2%) or working in commerce/industry (29.5%). Patients reported living in a detached house (58.3%), apartment (18.3%) or other (23.3%, including shelter, nursing home, prison or homeless). Contact with domestic animals was common (73.4%), especially dogs. Moreover, close contact with animals with leishmaniasis was described for 9/57 of patients. No clear seasonality was seen in respect to month of presentation of autochthonous primary episodes to healthcare, although March and June accounted for the most admissions or first consultations (12.0% and 11.5% of total, respectively).

Immunosuppressing conditions were present in 60.6% of patients. HIV infection/AIDS was reported in 53.5% of patients. Median CD4 cell count at time of diagnosis was 59.0/µlL (85.1% of patients had counts < 200/µl). Chronic pharmacologic immunosuppression for inflammatory diseases was reported in 10.8% of patients and other causes of immunosuppression included: solid organ transplant (*n* = 4), hematopoietic stem cell transplant (*n* = 1), solid organ malignancy (*n* = 4) and hematologic malignancy (*n* = 2). Immunosuppressing conditions and comorbidities of leishmaniasis patients are represented in Table 2.

**Table 2:** Immunosuppressing conditions and comorbidities of visceral leishmaniasis patients diagnosed in public hospitals in Mainland Portugal in 2010–2020

Immunosuppression, % (*n*)	
Yes	60.6
	(134/221)
Unknown/Not reported	8.1
	(18/221)
**HIV infection/AIDS**	
Yes, % (*n*)	53.5
	(108/202)
Median CD4 cell count, /µL (IQI)	59.0
	[21.5–127.0]
CD4 cell count < 200/µL, % (*n*)	85.1
	(86/101)
Detectable viral load, % (*n*)	65.6
	(63/96)
Median viral load, cp/mL (IQI)	80,000
	[220–631,400]
**Chronic pharmacologic immunosuppression, % (** ***n*** **)**	
Inflammatory/autoimmune diseases	10.8
	(21/194)
Anti-TNFα containing regimen	11.8
	(2/17)
Methotrexate ± corticosteroid	58.8
	(10/17)
Isolated corticosteroid	23.5
	(4/17)
Other^a^	5.9
	(1/17)
Solid organ transplant^b^	2.3
	(4/173)
**Chronic dysfunction/condition, % (** ***n*** **)**	
Diabetes mellitus	7.9
	(14/178)
Chronic kidney disease	12.5
	(22/178)
Chronic liver disease	13.3
	(24/181)
Chronic pulmonary disease	5.1
	(9/178)
Chronic heart failure	3.9
	(7/181)

*IQI *Interquartile interval, *HIV* Human immunodeficiency virus, *AIDS* Acquired Immunodeficiency syndrome, *TNF* Tumor Necrosis Factor

^a^Azathioprine + corticosteroid *n*  = 1, mycophenolate mofetil + corticosteroid *n*  = 1

^b^Kidney *n* = 3, liver *n* = 1

**Fig. 2:**
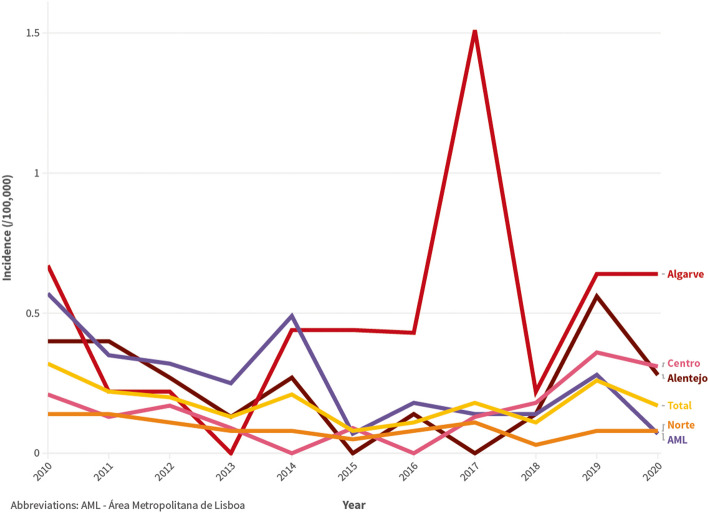
Yearly incidence of visceral leishmaniasis between 2010 and 2020 per 100,000 population, in Mainland Portugal and in each NUTS (Nomenclature of Territorial Units for Statistics) 2 region

**Fig. 3:**
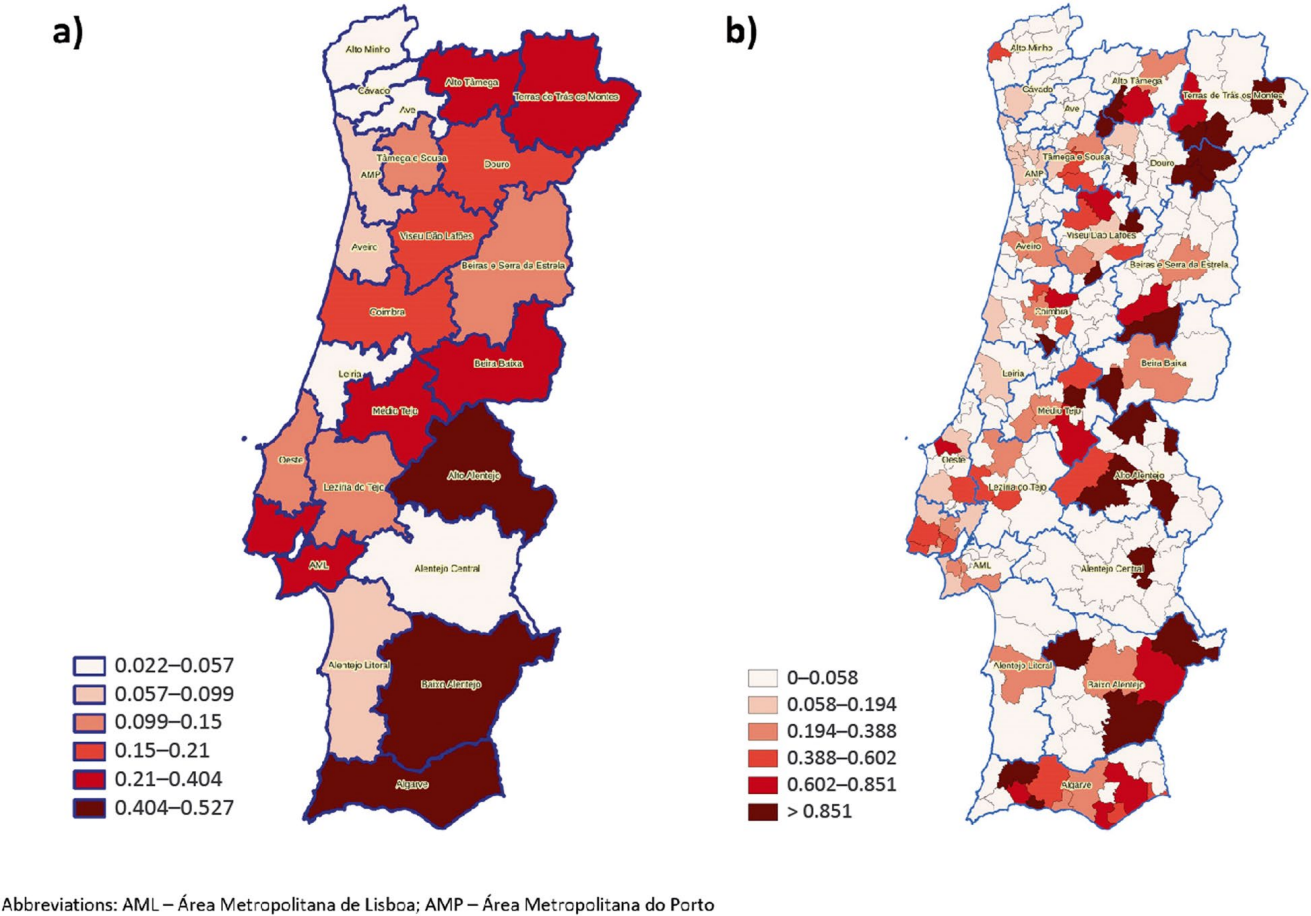
Mean annual incidence between 2010 and 2020, per 100,000 population, of visceral leishmaniasis by: **a** NUTS (Nomenclature of Territorial Units for Statistics) 3 region; **b** municipality

The estimated incidence of VL by year and by NUTS2 region is represented in Fig. 2. Globally, there was a significant decrease in incidence from 2010 to 2015–2016 (*P* = 0.001, CST); however, incidence subsequently increased and, in 2019–2020, it was significantly higher than in 2015–2016 (*P* = 0.030, CST). The Alentejo, Algarve and Centro regions presented increasing incidence in the 2017–2020 period. Figure 3 shows the incidence of VL by NUTS3 and municipality. The number of cases of VL diagnosed between 2010 and 2020, inclusively, and the incidence in this period by NUTS2 and NUTS3 region are also provided in Table 3. In the study period, the estimated incidence was highest in the Algarve (0.495 cases /100,000 population /year) and lowest in the Norte NUTS2 region (0.095 cases /100,000 population /year).

**Table 3:** Number of cases of visceral leishmaniasis diagnosed in public hospitals in Mainland Portugal between 2010 and 2020, inclusively, and estimated mean annual incidence in this period, per 100,000 population, by NUTS (Nomenclature of Territorial Units for Statistics) 2 and NUTS3 region

Region	Average population in 2011–2021^a^	Number of VL cases	VL mean annual incidence^b^	95% *CI*
Mainland Portugal	9,951,765	201	0.184	0.159–0.211
Norte	3,638,134	38	0.095	0.067–0.130
Alto Minho	238,051	1	0.038	0.001–0.213
Cávado	413,387	1	0.022	0.001–0.120
Ave	421,933	2	0.043	0.005–0.156
Área Metropolitana do Porto	1,747,876	15	0.078	0.044–0.129
Alto Tâmega	89,195	3	0.306	0.063–0.894
Tâmega e Sousa	420,776	6	0.130	0.048–0.282
Douro	194,516	4	0.187	0.051–0.479
Terras de Trás-os-Montes	112,399	5	0.404	0.131–0.944
Centro	2,277,497	38	0.152	0.107–0.208
Oeste	363,025	5	0.125	0.041–0.292
Região de Aveiro	368,898	4	0.099	0.027–0.252
Região de Coimbra	448,500	9	0.182	0.083–0.346
Região de Leiria	290,692	1	0.031	0.008–0.174
Viseu Dão Lafões	260,205	6	0.210	0.077–0.456
Beira Baixa	84,907	3	0.321	0.066–0.939
Médio Tejo	237,956	7	0.267	0.108–0.551
Beiras e Serra da Estrela	223,312	3	0.122	0.025–0.357
Área Metropolitana de Lisboa	2,846,042	81	0.259	0.206–0.322
Alentejo	730,917	19	0.236	0.142–0.369
Alentejo Litoral	97,183	1	0.094	0.002–0.521
Baixo Alentejo	120,777	7	0.527	0.212–1.086
Lezíria do Tejo	241,657	4	0.150	0.041–0.385
Alto Alentejo	111,714	6	0.488	0.179–1.063
Alentejo Central	159,585	1	0.057	0.001–0.317
Algarve	459,174	25	0.495	0.320–0.731

*VL* Visceral leishmaniasis, *CI* Confidence interval

^a^Arithmetic mean between the population size estimated in the National Census of 2011 and 2021

^b^Number of new cases per 100,000 population, per year. Based on the following formula: Incidence = (New Cases) / (Population x Timeframe)

### Clinical aspects

#### Clinical manifestations and laboratory alterations

Clinical presentation aspects of incident VL primary episodes are summarized in Table 4 globally and by group: children 5 years of age or younger (CU5), non-immunosuppressed adults and children over 5 years old (NISA), people living with HIV (PLWH) and non-HIV infected immunosuppressed adults (ISA).

**Table 4:** Clinical presentation characteristics of visceral leishmaniasis primary episodes, diagnosed in public hospitals in Mainland Portugal, and incident between 2010 and 2020, globally and by group

	Global	CU5	NISA	PLWH	ISA	*P*-value
**Number**	194	34	35	82	25	
**Median time from onset to presentation, weeks (IQI)**	4	2	5	4	4	0.01
	[1.5–11]	[1–3.5]	[1–19]	[2–14]	[1.75–9]	(*H* = 11.326, *df* = 3)
**Department of consultation/ward, % (** ***n*** **)**						
Infectious Diseases	40.9	0	18.2	66.7	37.5	< 0.001 (FET) (excluding CU5)
	(67/164)	(0/29)	(6/33)	(52/78)	(9/24)	
Internal Medicine	34.8	0	69.7	32.1	37.5	
	(57/164)	(0/29)	(23/33)	(25/78)	(9/24)	
Pediatrics	20.1	100	9.1	0	4.2	
	(33/164)	(29/29)	(3/33)	(0/78)	(1/24)	
Other	6.7	0	6.1	1.3	33.3	
	(11/164)	(0/29)	(2/33)	(1/78)	(8/24)	
**Hospital admission, % (** ***n*** **)**	94.5	100	96.9	91.1	95.8	0.363
	(155/164)	(29/29)	(31/32)	(72/79)	(23/24)	(FET)
Median duration, days (IQI)	20	13.5	22	22	28	0.04
	[12–36]	[9.0–17.75]	[11–49.5]	[12–45.75]	[17–34.5]	(*H* = 13.247, *df* = 3)
**Critical care, % (** ***n*** **)**	8.8	0	0	13.0	16.7	0.012
	(14/160)	(0/27)	(0/32)	(10/77)	(4/24)	(FET)
Median duration, days (IQI)	8.5	N/A	N/A	8.5	9.0	0.734
	[4.25–13.75]			[4.5–17.25]	[2.75–13.75]	(*U* = 0.116)
**Signs/Symptoms, % (** ***n*** **)**						
Splenomegaly	90.0	100	87.9	93.2	75.0	0.011
	(153/170)	(30/30)	(29/33)	(69/74)	(18/24)	(FET)
Fever	71.9	96.6	69.7	59.7	70.8	0.004
	(115/160)	(28/29)	(23/33)	(40/67)	(17/24)	(*χ* ^2^ = 13.254, *df* = 3)
Median highest value, ºC (IQI)	39.0	39.5	39.0	39.0	39.1	0.029
	[38.6–40.0]	[39.0–40.0]	[38.75–40.0]	[38.2–39.2]	[39.0–40.0]	(*H* = 9.046, *df* = 3)
Hepatomegaly	71.8	63.3	60.6	88.0	56.5	0.001
	(122/170)	(19/30)	(20/33)	(66/75)	(13/23)	(*χ* ^2^ = 15.826, *df* = 3)
Fatigue	69.8	29.6	81.3	75.0	76.2	< 0.001
	(113/162)	(8/27)	(26/32)	(54/72)	(16/21)	(*χ* ^2^ = 23.27, *df* = 3)
Anorexia	52.5	29.6	51.6	54.3	59.1	0.124
	(84/160)	(8/27)	(16/31)	(38/70)	(13/22)	(*χ* ^2^ = 5.755, *df* = 3)
Weight loss	49.7	13.0	56.7	57.6	36.4	0.001
	(75/151)	(3/23)	(17/30)	(38/66)	(8/22)	(*χ* ^2^ = 15.74, *df* = 3)
Gastrointestinal signs/symptoms	44.3	50.0	48.4	44.8	30.4	0.498
	(66/149)	(14/28)	(15/31)	(30/67)	(7/23)	(*χ* ^2^ = 2.377, *df* = 3)
Respiratory signs/symptoms	28.3	25.9	25.8	29.6	30.4	0.964
	(43/152)	(7/27)	(8/31)	(21/71)	(7/23)	(*χ* ^2^ = 0.279, *df* = 3)
Lymphadenopathy	23.0	14.3	29.0	27.5	13.0	0.272
	(37/161)	(4/28)	(9/31)	(19/69)	(3/23)	(*χ* ^2^ = 3.902, *df* = 3)
Skin/mucosal hemorrage^a^	22.7	10.7	15.6	27.1	33.3	0.135
	(35/154)	(3/28)	(5/32)	(19/70)	(8/24)	(*χ* ^2^ = 5.534, *df* = 3)
Neurological signs/symptoms	14.3	7.1	12.9	15.3	21.7	0.519
	(22/154)	(2/28)	(4/31)	(11/72)	(5/23)	(FET)
Peripheral edema	12.8	0	12.5	16.4	17.4	0.097
	(19/149)	(0/27)	(4/32)	(11/67)	(4/23)	(FET)
**Abdominal image obtained, % ( ** ***n*** **)** ^**b**^	96.9	96.6	97.0	96.0	100	1
	(155/160)	(28/29)	(32/33)	(72/75)	(23/23)	(FET)
**Analytical changes, % (** ***n*** **)**						
Anemia	98.9	100	97	100	100	0.533
	(173/175)	(30/30)	(32/33)	(77/77)	(25/25)	(FET)
Median lowest value, g/dL (IQI)	7.6	6.7	8.1	7.7	7.6	0.267
	[6.6–8.5]	[6.3–7.3]	[7.3–8.7]	[6.65–8.45]	[6.7–8.8]	(*H* = 2.642, *df* = 3)
Thrombocytopenia	90.2	90.0	87.5	90.9	88.0	0.930
	(157/174)	(27/30)	(28/32)	(70/77)	(22/25)	(FET)
Median lowest value, /µl (IQI)	64,000	76,000	54,000	69,000	39,000	0.026
	[36,500–112,000]	[47,500–120,250]	[31,000–106,500]	[44,000–116,500]	[16,000–92,000]	(*H* = 7.271, *df* = 2)
Leukopenia	88.3	65.5	87.1	94.8	96	< 0.001
	(143/162)	(19/29)	(27/31)	(73/77)	(24/25)	(FET)
Median lowest value, /µl (IQI)	2000	3450	1685	1765	1300	0.250
	[1272–2670]	[2597–4850]	[1025–2255]	[1200–2300]	[1077–1925]	(*H* = 2.769, *df* = 2)
C-reactive protein elevation	93.1	92.9	96.9	90.8	95.7	0.744
	(148/159)	(26/28)	(31/32)	(69/76)	(22/23)	(FET)
Median highest value, mg/L (IQI)	88.0	88.0	113.0	68.9	125.7	0.027
	[44.6–136.2]	[49.0–115.6]	[65.55–136.8]	[31.05–132.0]	[60.0–229.0]	(*H* = 7.254, *df* = 2)
Acute kidney failure	14.6	0	16.1	13.2	34.8	0.004
	(23/157)	(0/27)	(5/31)	(10/76)	(8/23)	(FET)
Liver failure/decompensated chronic liver disease	6.2	3.4	12.9	5.2	4.2	0.464
	(10/161)	(1/29)	(4/31)	(4/77)	(1/24)	(FET)
**Hemophagocytic lymphohistiocystosis, % (** ***n*** **)**	9.5	40.0	0	0	16.7	< 0.001
	(14/147)	(10/25)	(0/31)	(0/67)	(4/24)	(FET)
**Coinfection/superinfection, % (** ***n*** **)**	14.6	34.5	29.0	50.7	43.5	0.163
	(23/157)	(10/29)	(9/31)	(38/75)	(10/23)	(*χ* ^2^ = 5.122, *df* = 3)

* IQI* Interquartile interval, *FET* Fisher’s exact test, *CU5* Children 5 years of age or younger, *NISA* Non-immunosuppressed adults and children over 5 years old, *ISA* Non-HIV infected immunosuppressed adults, *PLWH* People living with HIV

^a^Lower gastrointestinal tract *n*  = 10, ecchymosis/hematoma/petechiae *n*  = 8, epistaxis *n*  = 7, hemoptysis *n*  = 7, gingival *n*  = 3, upper gastrointestinal tract *n*  = 1, vaginal *n*  = 1

^b^Ultrasonography 72.3% (112/155), computed tomography scan 53.5% (83/155)

Median time from onset of signs/symptoms to first presentation to healthcare was 4 weeks globally (IQI: 2–11) and was significantly different between groups (shorter in children under 5 years old, *P* = 0.010, *H* = 11.326, *df* = 3). In ISA, median time from start of immunosuppressive therapy to onset of signs/symptoms was 16 weeks (IQI: 12–66). Over 90% of patients in all groups were admitted as inpatients. Median duration of hospitalization was 20 days (IQI: 12–36) and was significantly different between groups: shortest in children [14] and longest in ISA [27] (*P* ≡ 0.040, KWT, *H* = 13.247, *df* = 3). Admission to critical care was only observed in ISA (16.7%) or in PLWH (13.0%). Fever was the most common presenting symptom (71.9%), followed by fatigue (69.8%), anorexia (52.5%) and weight loss (49.7%). Compared to NISA, fever was significantly less common in PLWH and more common in CU5, and the highest temperature was lower in PLWH and higher in CU5. Splenomegaly was detected in 90.0%, hepatomegaly in 71.8% and lymphadenopathy in 23.0%. Frequent laboratory abnormalities included: anemia (98.9%), thrombocytopenia (90.2%), leukopenia (88.3%), C-reactive protein (CRP) elevation (93.1%) and hepatic cytolysis or cholestasis (55.7%). Acute kidney failure was detected on admission in 14.6% of patients and was more common in ISA (*P* ≡ 0.004, FET). Criteria for HLH were met in 14 patients: 10 CU5 (40.0% of cases) and 4 in ISA (16.7%). Considering primary episodes and relapses, atypical presentations were diagnosed in 14 patients (8.5%), representing 12.8% of PLWH and 16.7% of ISA. Involvement was: colorectal (*n* = 6), duodenal/ileal (*n* = 7), gastric (*n* = 4), peritoneal (*n* = 1), pleural (*n* = 1), and bronchial (*n* = 1). Simultaneous involvement of the skin (with CL) was confirmed in 5.9% of patients. Coinfection/superinfection was detected in 42.4% of patients, without significant differences between groups, and was caused by the microbiological agents described in Supplementary Fig. 2. Respiratory and oropharyngeal/esophageal infections were the most common and *Candida* sp. and *Escherichia coli* were the most implicated microorganisms.

#### Diagnosis

Diagnosis, treatment, and outcome aspects of incident VL primary episodes are summarized in Table 5, globally and by group. Median time from presentation to diagnosis was 10 days (4.5–19.5) and was significantly different between groups: shortest in CU5 (5.5 days) and longest in ISA (17.5 days) (*P* ≡ 0.011, KWT, *H* = 11.192, *df* = 3). Samples most frequently used for direct diagnosis were: bone marrow (94.1%) and blood (25.0%). Techniques most often used in bone marrow samples were: microscopy (95.6%), PCR (41.6%), and culture (22.7%). Positivity rate was similar for PCR, microscopy, and culture (81.7%, 80.9% and 75.0%, respectively) and was not significantly different between groups. In blood samples, PCR was the technique most used for direct diagnosis (70.3%) and was positive in 73.1% of cases. In all cases in which *Leishmania* species identification was attempted and successful (*n* = 59), *L. donovani* complex was identified (by molecular biology techniques). Serologic techniques were used in 52.5% of patients, most commonly immunofluorescent antibody test (73.3%) and enzyme-linked immunosorbent assay (17.8%). Serology was positive in 82.9% of patients, ranging from 72.4% in PLWH to 92.3% in CU5, although this difference was not statistically significant (*P* ≡ 0.482, FET).

**Table 5:** Diagnosis, treatment, and outcome aspects of visceral leishmaniasis primary episodes, diagnosed in public hospitals in Mainland Portugal, and incident in the period between 2010 and 2020, globally and by group

	Global	CU5	NISA	PLWH	ISA	*P*-value
**Median time from presentation to diagnosis, days (IQI)**	10	5.5	11.0	10	17.5	0.011
	[4.5–19.5	[2.25–12.5]	[5.5–30.0]	[4.0–17.5]	[9.5–30.0]	(*H* = 11.192, *df* = 3)
**Samples used (direct diagnosis), % (** ***n*** **)**						
Bone marrow	94.1	96.4	100	93.4	82.6	0.058
	(160/170)	(27/28)	(34/34)	(71/76)	(19/23)	(FET)
Aspirate	91.4	81.5	97.1	93.0	89.5	0.158
	(139/152)	(22/27)	(33/34)	(66/71)	(17/19)	(FET)
Biopsy	50.7	59.3	50.0	46.5	57.9	0.639
	(77/152)	(16/27)	(17/34)	(33/71)	(11/19)	(*χ* ^2^ = 1.692, *df* = 3)
Blood	25.0	34.8	17.9	13.7	13.6	0.166
	(37/148)	(8/23)	(5/28)	(10/73)	(3/22)	(FET)
**Technique used in bone marrow sample, % (** ***n*** **)**						
Microscopy	95.6	92.3	96.7	97.3	95.5	0.609
	(152/159)	(24/26)	(29/30)	(71/73)	(21/22)	(FET)
Positive result	80.9	66.7	75.9	83.1	90.5	0.193
	(123/152)	(16/24)	(22/29)	(59/71)	(19/21)	(FET)
Polymerase chain reaction	41.6	60.9	32.3	38.2	47.4	0.158
	(62/149)	(14/23)	(10/31)	(26/68)	(9/19)	(*χ* ^2^ = 5.194, *df* = 3)
Positive result	81.7	78.6	60.0	87.5	88.9	0.315
	(49/60)	(11/14)	(6/10)	(21/24)	(8/9)	(FET)
Culture	22.7	9.5	20.0	29.2	11.8	0.200
	(32/141)	(2/21)	(6/30)	(19/65)	(2/17)	(FET)
**Technique used in blood sample, % (** ***n*** **)**						
Polymerase chain reaction	17.8	34.8	17.9	13.7	13.6	0.166
	(26/146)	(8/23)	(5/28)	(10/73)	(3/22)	(FET)
Positive result	73.1	87.5	60.0	80.0	33.3	0.321
	(19/26)	(7/8)	(3/5)	(8/10)	(1/3)	(FET)
**Serology, % (** ***n*** **)**						
Yes^a^	52.5	54.2	69.7	41.4	50.0	0.063
	(83/158)	(13/24)	(23/33)	(29/70)	(11/22)	(*χ* ^2^ = 7.288, *df* = 3)
Positive result	82.9	92.3	86.4	72.4	81.8	0.482
	(68/82)	(12/13)	(19/22)	(21/29)	(9/11)	(FET)
**Samples sent to reference laboratory, % (** ***n*** **)**	40.0	55.0	44.0	31.1	47.4	0.216
	(50/125)	(11/20)	(11/25)	(19/61)	(9/19)	(*χ* ^2^ = 4.463, *df* = 3)
**Treatment of primary episode, % (** ***n*** **)**						
Yes	99.4	100	100	98.7	100	1
	(161/162)	(27/27)	(32/32)	(78/79)	(24/24)	(FET)
Median time from diagnosis to treatment, days (IQI)	0	0	0	0	0	0.787
	[0–1]	[0–1]	[0–0.2]	[0–0.2]	[0–4.75]	(*H* = 1.060, *df* = 3)
Median duration of treatment, days (IQI)	21	21	21	21	24	0.031
	[10–38]	[7.75–21.0]	[10.0–21.0]	[10.0–38.0]	[11.0–38.0]	(*H* = 9.894, *df* = 3)
Liposomal amphotericin B monotherapy	98.8	92.6	100	100	100	1
	(158/160)	(25/27)	(32/32)	(77/77)	(24/24)	(FET)
Side effects	30.5	9.5	42.3	25.0	45.5	0.025
	(40/131)	(2/21)	(11/26)	(15/60)	(10/22)	(*χ* ^2^ = 9.365, *df* = 3)
**Outcome of treatment, % (** ***n*** **)**						
Median time to defervescence, days (IQI)	3	2	3	3	6	0.008
	[1.75–5]	[1.0–3.0]	[1.0–3.0]	[2.0–5.5]	[2.5–10.5]	(*H* = 11.823, *df* = 3)
Improvement at 7 days	88.6	100	96.6	87.1	69.6	0.003
	(132/149)	(27/27)	(28/29)	(61/70)	(16/23)	(FET)
Improvement at 30 days	96.4	100	100	98.5	82.6	0.008
	(135/140)	(25/25)	(27/27)	(64/65)	(19/23)	(FET)
Switch of treatment/retreatment^b^	3.9	3.6	3.2	2.7	8.3	0.583
	(6/153)	(1/28)	(1/31)	(2/73)	(2/24)	(FET)
Death in current episode	4.3	0	0	5.2	13.0	0.066
	(7/163)	(0/30)	(0/33)	(4/77)	(3/23)	(FET)
**Relapse**						
Rate (episodes/patient-year)	0.112	0	0	0.175	0.147	0.578
Median time to first relapse, months (IQI)	12	N/A	N/A	15	6	0.009
	[7.25–33.5]			[9.5–36.0]	[5.0–11.0]	(*U* = 48.0)
**Secondary profilaxis initiated** ^**c**^ **, % (** ***n*** **)**	29.7	4.3	0	54.9	16.7	< 0.001
	(44/148)	(1/23)	(0/30)	(39/71)	(4/24)	(*χ* ^2^ = 43.327, *df* = 3)
**Follow-up in consultation, % (** ***n*** **)**						
Yes	93.3	92.3	96.9	91.5	95.2	0.813
	(140/150)	(24/26)	(31/32)	(65/71)	(20/21)	(FET)
Cure test performed^d^	16.9	13.6	3.4	24.2	15.8	0.080
	(23/136)	(3/22)	(1/29)	(16/66)	(3/19)	(FET)
**Notification of case to SINAVE, % (** ***n*** **)**	49.7	75.8	50.0	40.0	50.0	0.006
	(92/185)	(25/33)	(18/36)	(36/90)	(13/26)	(*χ* ^2^ = 12.353, *df* = 3)

* IQI* Interquartile interval, *FET* Fisher’s exact test, *CU5* Children 5 years of age or younger, *NISA* Non-immunosuppressed adults and children over 5 years old, *ISA* non-HIV infected immunosuppressed adults, *PLWH* People living with HIV, *SINAVE* Sistema Nacional de Vigilância Epidemiológica

^a^Immunofluorescent antibody test (33/45), Enzyme-linked immunosorbent assay (8/45), Western Blot (5/45), k39 rapid diagnostic test (2/45)

^b^Due to side effects *n*  = 3, Due to non improval *n*  = 3

^c^Liposomal amphotericin B *n*  = 42, Miltefosine *n*  = 1, unknown *n*  = 1

^d^Bone marrow microscopy *n*  = 12, PCR in blood *n*  = 10

#### Treatment and outcome

In most cases, treatment was initiated on the same day of diagnosis (median time 0 days, IQI: 0–1). Liposomal amphotericin B (LAmB) was used for primary treatment in 98.8% of cases and meglumine antimoniate for the rest (*n* = 2, both CU5). Side effects were reported globally in 30.5% of patients (*n* = 40) and were significantly less common in CU5 (*P* ≡ 0.025, CST, *χ* ^2^ = 9.365, *df* = 3). The reported side effects included: acute kidney injury and/or hypokalemia (19.8%, *n* = 26), hepatotoxicity (*n* = 4), vomiting and/or diarrhea (*n* = 4), fever/shivering (*n* = 3), myalgia (*n* = 2), anaphylaxis (*n* = 1). In PLWH, antiretroviral therapy was initiated or reinitiated in 48.6% of patients; one case of paradoxical immune reconstitution inflammatory syndrome was documented. In ISA, withdrawal of immunosuppressive drugs or reduction of dose was done in 66.7% of cases. Median time to defervescence after initiation of anti-*Leishmania* therapy was 3.0 days (IQI: 1.75–5) and was significantly shorter for CU5 and longer for ISA (*P* ≡ 0.008, KWT, *H* = 11.823, *df* = 3). Improvement by day 7 after initiation of anti-*Leishmania* therapy was documented in 88.6% of cases, ranging from 69.6% in ISA to 87.1% in PLWH and 100% in CU5 (*P* ≡ 0.003, FET). Improvement by day 30 after initiation of therapy was documented in 96.4% of patients and was over 95% in all groups except ISA (82.6%, *P* ≡ 0.008, FET). Death occurred in seven cases (4.3%): four PLWH (5.2%) and three ISA (13.0%). Secondary prophylaxis was implemented in 54.9% of PLWH, but in only 16.7% of ISA (*P* = 0.001, *χ* ^2^ = 10.599, *df* = 1); drugs used for prophylaxis were LAmB (97.7%) and miltefosine (2.3%). Cure tests were performed for 16.9% of patients, especially PLWH, and median time to cure test was 6.1 weeks after completing primary treatment (IQI: 3.25–23.5).

#### Relapses

**Fig. 4:**
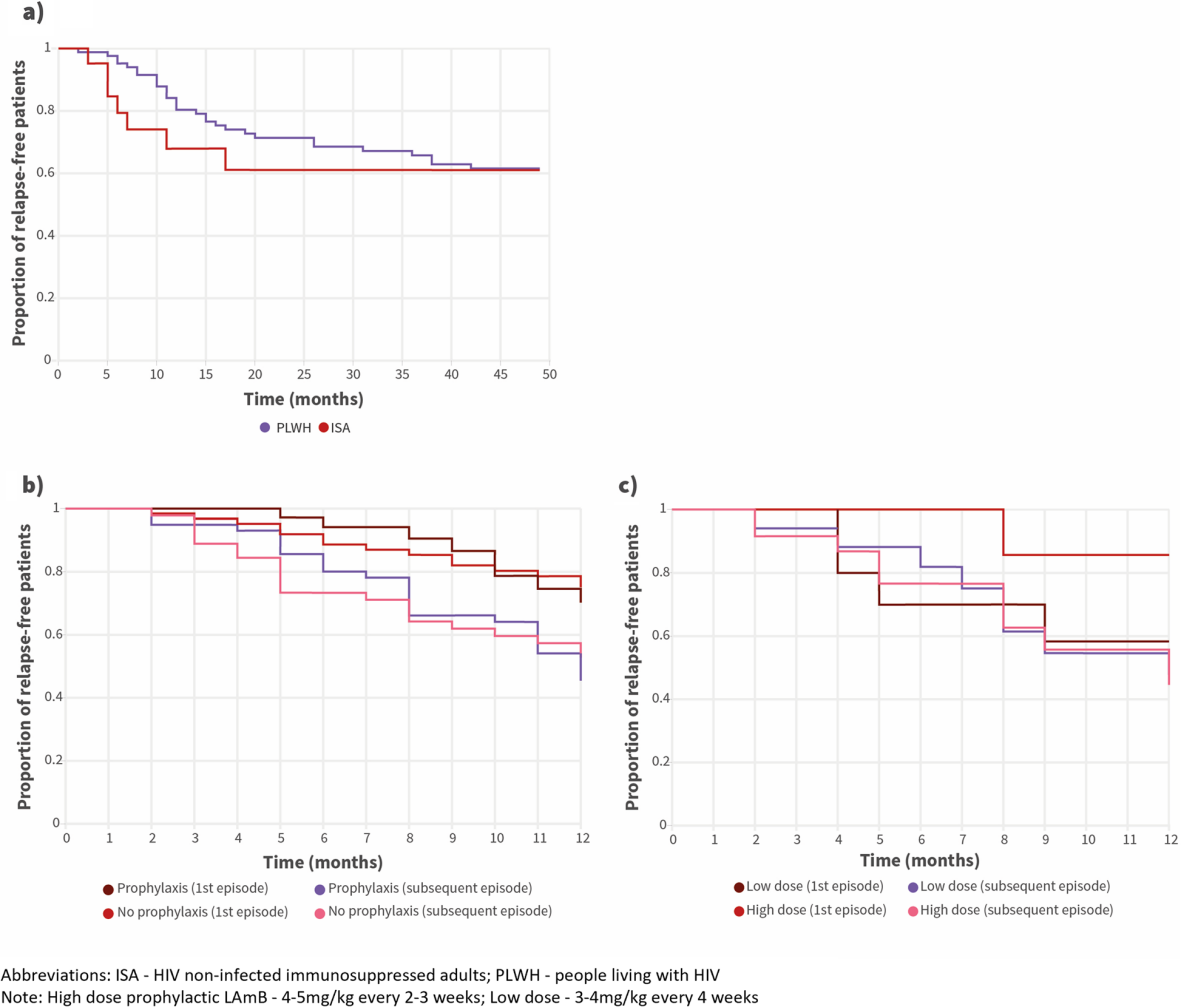
Relapse-free survival of visceral leishmaniasis patients: **a** according to group; **b** according to use of prophylaxis and primary/subsequent episode; **c** according to dose of liposomal amphotericin B used and primary/subsequent episode

In total, there were 151 episodes of relapse in the study period, affecting 61 patients. The number of relapses per patient ranged from 1 to 9. Relapses were documented only in PLWH and in ISA, at a similar rate: 0.175 and 0.147 episodes per patient-year, respectively (*P* ≡ 0.578, CST). Relapse-free survival was significantly higher for PLWH than for ISA at nine months follow-up (*P* ≡ 0.023, *χ* ^2^ = 5.203, *df* = 1) but not at 48 months (*P* ≡ 0.453, *χ* ^2^ = 0.562, df = 1) (Fig. 4a). Relapse-free survival was slightly higher for patients on any prophylaxis in the first 10 months after a primary VL episode or a relapse (Fig. 4b), but this difference did not reach statistical significance (*P* ≡ 0.396, *χ* ^2^ = 0.720, *df* = 1 for primary episodes; *P* ≡ 0.674, *χ* ^2^ = 0.177, *df* = 1, for relapses). Rate of relapse was significantly higher in the 12 months after a relapse than after a primary episode, either with or without prophylaxis (*P* ≡ 0.023, *χ* ^2^ = 5.195, *df* = 1; *P* ≡ 0.012, *χ* ^2^ = 6.364, *df =* 1, respectively). Use of LAmB for prophylaxis at doses of 4–5 mg/kg every 2–3 weeks was associated with significantly higher relapse-free survival at 12 months than doses of 3–4 mg/kg every 4 weeks, for primary episodes (*P* ≡ 0.048, *χ* ^2^ = 3.893, *df* = 1), but not for relapses (*P* ≡ 0.862, *χ* ^2^ = 0.030, *df* = 1) (Fig. 4c).

In relapse cases, compared to primary episodes (in PLWH or ISA), time from onset to presentation was significantly shorter (median 3.0 vs. 4.0 weeks, *P* ≡ 0.030, *U* = 3377.0). Drugs used for secondary prophylaxis after a relapse included LAmB (80.5%), miltefosine (6.5%), and LAmB + miltefosine (6.5%). Outcome of treatment of VL relapses according to drug used is represented in Table 6. The percentage of episodes with improvement was higher for combination therapy at 7 (90.0 vs. 80.8%) and 30 days (93.3 vs. 83.5%) after initiation of therapy, but this difference was not statistically significant (*P* ≡ 0.238, *χ* ^2^ = 1.395, *df* = 1; *P* ≡ 0.235, FET, respectively). Subsequent relapse-free survival after a relapse was not significantly different for patients treated with monotherapy or combination therapy (*P* ≡ 0.816, *χ* ^2^ = 0.054, *df* = 1). Side effects were less commonly reported for LAmB (39.4%), compared to miltefosine (55.6%) or meglumine antimoniate (71.4%).

**Table 6:** Outcome of treatment of episodes of relapse of visceral leishmaniasis, diagnosed in public hospitals in Mainland Portugal in 2010–2020, according to drug or combination of drugs used

	Total	Monotherapy	LAmB	Miltefosine	Meglumine antimoniate	Combination	LAmB + Miltefosine	LAmB + Paromomycin	Others	*P*-value (monotherapy vs. combination)	*P*-value (LAmB vs. LAmB + miltefosine)	*P*-value (LAmB vs. miltefosine vs. meglumine)
**Number, %** ***(n*** **)**	100	77.3	63.1	6.4	7.8	22.7	17.7	2.1	2.8			
	(141/141)	(109/141)	(89/141)	(9/141)	(11/141)	(32/141)	(25/141)	(3/141)	(4/141)^a^			
**Side effects, %** ***(n*** **)**	44.0	43.7	39.4	55.6	71.4	44.8	37.5	100	50.0	0.914	0.866	0.169
	(51/116)	(38/87)	(28/71)^b^	(5/9)^c^	(5/7)^d^	(13/29)	(9/24)^e^	(3/3)^f^	(1/2)	(*χ* ^2^ = 0.012, *df* = 1)	(*χ* ^2^ = 0.028, *df* = 1)	(FET)
**Result, %** ***(n*** **)**												
Improvement at 7 days	82.8	80.8	81.4	77.8	77.8	90.0	87.5	100	100	0.238	0.760	0.798
	(111/134)	(84/104)	(70/86)	(7/9)	(7/9)	(27/30)	(21/24)	(3/3)	(3/3)	(*χ* ^2^ = 1.395, *df* = 1)	(FET)	(FET)
Improvement at 30 days	86.2	83.5	84.0	75.0	87.5	93.3	91.7	100	100	0.235	0.507	0.852
	(106/123)	(76/91)	(63/75)	(6/8)	(7/8)	(28/30)	(22/24)	(3/3)	(3/3)	(FET)	(FET)	(FET)
**Subsequent relapse**												
Median time to relapse, months (IQI)	11.0	11.0	11.0	8.5	11.0	11.0	11.0	14.5	12.0	0.789	0.519	0.536
	[7.0–19.0]	[6.5–19.0]	[5.0–19.5]	[4.75–14.0]	[8.25–17.5]	[6.5–15.0]	[5.0–13.5]	[11.0–18.0]	[8.0–16.0]	(*U* = 696.5)	(*U* = 403,5)	(*H* = 1.248, *df* = 2)

*IQI* Interquartile interval, *LAmB* Liposomal amphotericin B

^a^LAmB + Meglumine + Voriconazol *n* = 1; LAmB + Paromomycin + Miltefosine *n* = 1; Meglumine + Miltefosine *n* = 1; Meglumine + Paromomycin *n* = 1

^b^Renal dysfunction *n* = 20, Fever/shivering *n* = 2

^c^Diarrhea *n* = 5

^d^Pancreatitis *n* = 5, cardiac toxicity *n* = 1

^e^Renal dysfunction *n* = 6, diarrhea/vomiting *n* = 3

^f^Renal dysfunction *n* = 2, Ototoxicity *n* = 1

#### Notification of cases and regional differences

Only 49.7% of incident VL cases in 2010–2020 were notified to the National Epidemiologic Surveillance System; cases in CU5 were significantly more notified (75.8%, *P* ≡ 0.006, *χ* ^2^ = 12.353, *df* = 3). The percentage of cases notified was significantly different according to the region of the hospital: Norte 45.7%, Centro 69.7%, AML 44.0%, Alentejo 81.8%, and Algarve 70.8% (*P* = 0.007, *χ* ^2^ = 14.106, *df* = 4); notification was not significantly different for patients admitted to Internal Medicine (45.6%) or Infectious Diseases departments (46.3%, *P* = 0.942, *χ* ^2^ = 0.005, *df* = 1).

The main regional differences in presentation and management of VL are summarized in Table 7. The Alentejo was the region with a lower percentage of cases in PLWH/ISA and a higher percentage in CU5. In the Algarve and the Alentejo regions more patients were admitted to Internal Medicine vs. Infectious Diseases departments and time from presentation to diagnosis was longer in these regions. Use of serology for diagnosis was more common in the Alentejo, and less common in the Algarve.

**Table 7:** Presentation and management of incident visceral leishmaniasis cases diagnosed in public hospitals in Mainland Portugal in 2010–2020, by NUTS2 region of residence: Norte, Centro, Área Metropolitana de Lisboa, Alentejo and Algarve

	Global	Norte	Centro	AML	Alentejo	Algarve	*P*-value
**Number**, ***n***	194	37	36	79	18	24	
Children under 5 years old, % (*n)*	18.9	20.6	17.4	16.7	35.3	13	0.449
	(33/175)	(7/34)	(4/23)	(13/78)	(6/17)	(3/23)	(FET)
Immunosuppressed patients, % (*n)*	61.1	50.0	65.2	69.2	35.3	65.2	0.060
	(107/175)	(17/34)	(15/23)	(54/78)	(6/17)	(15/23)	(*χ* ^2^ = 9.027, *df* = 4)
PLWH, % (*n)*	46.9	29.4	47.8	61.5	17.6	43.5	0.002
	(82/175)	(10/34)	(11/23)	(48/78)	(3/17)	(10/23)	(*χ* ^2^ = 16.846, *df* = 4)
**Median time from onset to presentation, weeks (IQI)**	4.0	3.0	8.0	4.0	2.0	2.0	0.086
	[1.5–11.0]	[1.0–12.0]	[2.5–17.0]	[2.0–10.0]	[1.0–4.0]	[1.0–5.5]	(*H* = 8.152, *df* = 4)
**Department of consultation/ward, % (** ***n*** **)**							
Internal Medicine	43.5	34.6	35.3	33.3	62.5	85.0	< 0.001 (FET)
	(57/131)	(9/26)	(6/17)	(20/60)	(5/8)	(17/20)	
Infectious Diseases	51.1	46.2	58.8	66.7	25.0	15.0	
	(67/131)	(12/26)	(10/17)	(40/60)	(2/8)	(3/20)	
**Hospital admission, % (** ***n*** **)**	94.5	87.1	89.5	97.4	93.3	100	0.094
	(155/164)	(27/31)	(17/19)	(74/76)	(14/15)	(23/23)	(FET)
Median duration, days (IQI)	20.0	16.5	29.0	20.0	16.0	29.0	0.290
	[12.0–36.0]	[7.0–35.25]	[13.0–41.0]	[11.75–32.75]	[11.0–23.0]	[14.0–56.0]	(*H* = 4.973, *df* = 4)
**Median time from presentation to diagnosis, days (IQI)**	10.0	12.0	8.0	8.0	15.0	20.0	0.004
	[4.5–19.5]	[2.5–26.5]	[2.25–20.75]	[4.0–15.0]	[7.0–50.0]	[8.0–60.0]	(*H* = 15.413, *df* = 4)
**Samples used (direct diagnosis), % (** ***n*** **)**							
Bone marrow	94.1	87.9	88.0	98.7	92.9	95.7	0.054
	(160/170)	(29/33)	(22/25)	(74/75)	(13/14)	(22/23)	(FET)
Blood	17.8	27.6	5.9	13.6	41.7	13.6	0.066
	(26/146)	(8/29)	(1/17)	(9/66)	(5/12)	(3/22)	(FET)
**Technique used in bone marrow sample, % (** ***n*** **)**							
Microscopy	95.6	92.6	91.7	95.9	100	100	0.609
	(152/159)	(25/27)	(22/24)	(70/73)	(13/13)	(22/22)	(FET)
PCR	41.6	39.3	43.5	44.6	76.9	10.0	0.004
	(62/149)	(11/28)	(10/23)	(29/65)	(10/13)	(2/20)	(*χ* ^2^ = 15.234, *df* = 4)
Culture	22.7	46.4	22.7	35.5	22.2	0	0.002
	(32/141)	(13/28)	(5/22)	(22/62)	(2/9)	(0/20)	(FET)
**Serology, % (** ***n*** **)**	52.5	46.9	60.0	59.1	69.2	22.7	0.022
	(83/158)	(15/32)	(15/25)	(39/66)	(9/13)	(5/22)	(*χ* ^2^ = 11.400, *df* = 4)
**Samples sent to reference laboratory, % (** ***n*** **)**	40.0	33.3	11.1	46.3	80.0	27.3	0.014
	(50/125)	(10/30)	(1/9)	(25/54)	(8/10)	(6/22)	(FET)
**Treatment of primary episode, % (** ***n*** **)**							
Median tme from diagnosis to treatment, days (IQI)	0.0	0.0	1.0	0.0	0.0	0.0	0.001
	[0.0–1.0]	[0.0–3.0]	[0.0–2.5]	[0.0–5.0]	[0.0–1.75]	[0.0–0.0]	(H = 18.384, *df* = 4)
Median duration of treatment, days (IQI)	21.0	21.0	21.0	21.0	21.0	21.0	0.954
	[10.0–38.0]	[10.0–38.0]	[18.0–28.0]	[10.0–38.0]	[11.0–38.0]	[10.0–38.0]	(H = 0.681, *df* = 4)
**Outcome of treatment, % (** ***n*** **)**							
Improvement at 7 days	88.6	80.8	76.5	91.4	100	91.3	0.176
	(132/149)	(21/26)	(13/17)	(64/70)	(13/13)	(21/23)	(FET)
Improvement at 30 days	96.4	96.2	92.9	98.5	90.9	95.7	0.306
	(135/140)	(25/26)	(13/14)	(65/66)	(10/11)	(22/23)	(FET)
**Follow-up in consultation, % (** ***n*** **)**							
Yes	93.3	100	81.3	91.5	92.9	100	0.880
	(140/150)	(26/26)	(13/16)	(65/71)	(13/14)	(23/23)	(FET)
Median time to first consultation, days (IQI)	15.5	12.0	21.5	12.0	13.0	30.0	0.004
	[7.0–30.0]	[7.0–28]	[7.75–43.75]	[5.5–27.0]	[7.0–16.0]	[18.0–55.0]	(*H* = 15.318, *df* = 4)
Cure test performed	16.9	28.6	0	16.1	10.0	17.4	0.236
	(23/136)	(8/28)	(0/13)	(10/62)	(1/10)	(4/23)	(FET)

*AML* Área Metropolitana de Lisboa, *IQI* Interquartile interval, *FET* Fisher’s exact test, *PCR* Polymerase chain reaction, *PLWH* People living with HIV

### Associations in VL

In univariate analysis, non-improvement at day 7 after initiation of anti-*Leishmania* therapy for primary treatment of VL was associated with male sex, immunosuppression, chronic organ dysfunction, renal failure at admission, severe leukopenia (< 1500/µl), coinfection/superinfection and CRP level over 100 (Table 8a). However, in multivariate analysis, CRP level over 100 was the only statistically significant factor.

**Table 8:** Potential factors for non-improvement at 7 days after starting treatment and for non-reporting of primary cases newly diagnosed between 2010 and 2020 in public hospitals in Mainland Portugal, according to logistic regression models to estimate crude and adjusted odds ratio values

a)	Potential Risk Factor	Univariate			Multivariate		
		% in Sample	Crude OR	95% CI	Adjusted OR	95% CI	*P*-value
Non-improvement at 7 days	Male sex	68.5	3.88	[0.85–17.88]	2.66	[0.49–14.41]	0.257
	Immunosuppressed	62.4	11.36	[1.47–90.91]	5.71	[0.65–50.0]	0.115
	Chronic organ dysfunction	24.6	2.72	[0.93–7.94]	2.79	[0.65–12.05]	0.168
	Acute kidney injury	15.2	3.82	[1.24–11.76]	1.37	[0.32–5.92]	0.670
	Leucocyte count < 1500/µl	37.7	3.86	[1.24–12.01]	2.47	[0.62–9.76]	0.199
	CRP > 100 mg/L	43.6	2.69	[0.93–7.75]	5.18	[1.19–22.22]	**0.028**
	Coinfection/superinfection	41.5	3.17	[1.03–9.80]	1.62	[0.32–5.95]	0.468
	Constant				77.713		< 0.001
	Hosmer and Lemeshow Test				0.758		
**b)**	Potential Risk Factor	Univariate			Multivariate		
		% in Sample	Crude *OR*	95% *CI*	Adjusted *OR*	95% *CI*	*P*-value
Non-reporting	Age > 5 years old	17.5	2.78	[1.22–6.32]	4.07	[1.19–13.93]	**0.026**
	Immunosuppressed	60.8	2.11	[1.14–3.92]	1.15	[0.46–2.87]	0.758
	Region of hospital AML or Norte	64.9	3.22	[1.71–6.09]	3.91	[1.74–8.75]	**< 0.001**
	Secondary center	50.3	2.02	[1.13–3.60]	2.09	[1.03–4.27]	**0.042**
	Non-improvement at 7 days	11.4	1.66	[0.60–4.63]	1.56	[0.50–4.86]	0.446
	Constant				0.345		0.310
	Hosmer and Lemeshow Test				0.354		

*OR* Odds ratio, *CI* Confidence interval, *CRP* C-reactive protein, *AML* Área Metropolitana de Lisboa

Non-reporting of a VL case was associated in univariate analysis with age over 5 years old, immunosuppressed status, admission to a hospital located in the Norte or AML region and admission to a secondary center. In multivariate analysis, age over 5 years old, admission to a hospital located in the Norte or AML region and admission to a secondary center remained significant (Table 8b).

## Discussion

The present study raises the attention to the ongoing burden of VL in Portugal, especially in children and in PLWH and other immunosuppressed patients. Until 2015–16, the calculated national incidence seems to follow the decreasing trend observed in a study respective to the 1999–2009 period [12], but in more recent years incidence seems to be increasing, driven mostly by increasing regional incidence in the Centro, Algarve, and Alentejo NUTS2 regions. An increasing absolute number of cases in the Algarve region had already been noted in previous reports [20]. Some NUTS3 regions with highest incidence in the 2010–2020 period largely overlap the districts where canine seroprevalence was estimated as highest in a recent study [21], namely Terras de Trás-os-Montes, Beiras e Serra da Estrela, Beira Baixa, and Alto Alentejo. Similarly, the Ave, Cávado and Alto Minho regions were expected to be lower incidence areas, according to canine seroprevalence [20] and previous human case report data [11]. Regions such as the AML and the Douro region, traditionally recognized as endemic foci of disease [22] remain so, despite showing an intermediate incidence in the present study. PLWH continue to represent the major group of VL cases, other immunosuppressed patients represent an increasing percentage compared to the 1999–2009 period (12.3 vs. 6.5%) [12]. Children 10 years old or younger, in contrast, represented only 16.3% of cases vs. 30.4% in the previous decade [12]. This shift in affected populations could represent a reduced risk of ongoing active primary transmission of *Leishmania* and an increased contribution of reactivating infection in a growing population of immunosuppressed adults. Increasing incidence in more recent years could be related to changing environmental conditions favoring prolonged vector survival and geographical expansion, as seen in other areas in Europe, including in Northern Spain [23] and as expected by modelling [24]. Additionally, the changing epidemiology of the HIV pandemic could help explain the evolution of VL incidence since 2000, taking into consideration progressive decreasing incidence of new diagnosis of HIV infection and of AIDS in Portugal [25]. The Algarve is the region with the second highest HIV infection incidence in recent years, after the AML [25]; approximately 85% of cases of VL diagnosed in PLWH occurred in the setting of CD4 cell counts < 200/µl and, according to the most current data, 37.9% of PLWH are still diagnosed at this stage [25]. In summary, these findings suggest that, in the Mediterranean context, control of HIV infection, including early diagnosis and prevention of transmission is a cornerstone in controlling VL. Predominance of male sex has been described previously in Portugal and in other Mediterranean countries [10] and has been attributed to biological factors, besides sociocultural determinants [26].

Imported disease still represents a minority of cases in Portugal, opposed to other European endemic countries such as (metropolitan) France [27]; however, this could be expected to change in upcoming years, in relation with increasing migration from VL endemic countries such as Brazil, India and Nepal [28]. Since no systematic clinical screening program is implemented in migrant populations in Portugal, leishmaniasis cases could go unnoticed and translate into an underestimation of imported cases. On the other hand, even though migrants represent only 5.2% of the Portuguese national population [28], they represented 19.1% of VL cases; these cases were mostly autochthonous, in people born in non-endemic sub-Saharan African countries. This disproportionally high burden in migrant populations suggests their increased vulnerability to locally acquired infections, besides the risk for imported disease. Additionally, a higher percentage of homelessness and of unemployment was seen in VL cases compared to the national value (22.2 vs. 6.6%) [29], reinforcing leishmaniasis as a disease of neglect and of deprived settings.

The fact that immunosuppressed patients represent an increasing share of VL cases, including in the setting of use of methotrexate, and/or anti-TNFα drugs, raises the attention to the role that screening prior to starting these therapies could have in preventing symptomatic primary *Leishmania* infection or reactivation. Currently, there is no consensus on the indications for screening, nor on the techniques that should be used and how to define asymptomatic infection [30]. Management of asymptomatic infection, when detected, is currently based on clinical monitoring and no treatment strategies have been adequately studied [31]. These gaps should be addressed.

Regarding VL, even though the clinical findings have largely been already described in the Mediterranean context, including in Portugal [12], the present study contributes to reinforce dissimilarities in presentation in the different groups included. In children five years of age or younger, compared to older patients, the presentation was more abrupt, and time from onset of signs/symptoms to presentation to healthcare was shorter. Fever was more commonly reported, and the median highest temperature was higher. Splenomegaly was present in all cases, but weight loss, anorexia and fatigue were less frequent. Criteria for HLH were met in 40% of children – this percentage is somewhat higher than observed in studies performed in the Mediterranean [32] and Brazilian [33] contexts (possibly representing statistical variation associated with the small sample size in all of these studies) and highlights the need to rule out VL in all children presenting with HLH in endemic settings.

In PLWH, compared to non-immunosuppressed adults, fever was less common, and lower grade, and hepatomegaly was more common. Maximal CRP elevation was lower. Bacterial, fungal and/or viral coinfection was most common.

ISA were more frequently admitted to Internal Medicine wards, but also to other specialties according to their underlying conditions, reinforcing the growing need to recognize leishmaniasis in transplant, oncology, and hematology settings. Diagnostic delay was especially pronounced in ISA, in whom VL is less commonly considered in the differential diagnostic list. This group presented more severe disease, with longer hospital stays, more frequent admission to critical care, more frequent kidney failure, lower median platelet counts, more frequent skin/mucosal hemorrhage, and higher percentage of deaths. Of note 4/24 patients presented HLH, which has been rarely reported in adults with VL; however, VL still represents a considerable share of all adult HLH cases [34].

A high rate (8.5%) of atypical presentations was documented, especially with involvement of the gastrointestinal tract. Current knowledge of these forms of disease is limited to case reports and small series [35, 36], and suggests that they do not have a poorer prognosis or response to treatment than classic VL (in patients with similar immune status), but they could pose a diagnostic challenge in patients in whom other findings such as pancytopenia, splenomegaly or fever are absent.

Regarding diagnostic techniques, microscopy of bone marrow was preferred, even though European guidelines suggest a first approach using serology [17], probably reflecting unavailability in many centers and, on the other hand, the fact that bone marrow biopsy/aspirate allows investigation of alternative diagnoses. Use of PCR increased compared to the previous 10-year period (41.6 vs. 25.1%) [12]; in particular, use of PCR in blood has emerged as a less invasive alternative, with a reasonable positivity rate, both in the present and in previous studies [37]. In all cases of VL in which identification of complex was performed, *L. donovani* complex was identified; efforts for identification to the species level should be intensified, taking into account the increasing migrant population from South Asia [35] and the risk of introduction of anthroponotic and clinically distinct *L. donovani* sensu stricto (s.s.). Phlebotomine vectors for *L. infantum*, widely distributed across Portugal, are also permissive for *L. donovani* s.s [38]. This species has already been documented in Cyprus in humans and dogs [39] and hybrids between *L. infantum* and *L. donovani* have been demonstrated in Turkey [40].

Contrasting with the 1999–2009 period, when meglumine antimoniate was frequently used [12], in the present study LAmB was almost the only drug used to treat primary episodes of VL, in accordance with European guidelines [36]. There are no randomized clinical trials to support the use of combination therapy (LamB + miltefosine) in the Mediterranean setting, although this strategy has been studied in PLWH in South Asia, where *L. donovani* s.s. is endemic, revealing significantly higher relapse-free survival at day 210, compared to LAmB monotherapy [41]. In the present study, clinical response was comparable to described in the literature in Europe (cure rates > 90%) [42], but differed among groups: faster and greater in CU5, evidenced by shorter interval to defervesce and higher percentage of patients with clinical improvement by days 7 and 30 after initiating LAmB. ISA showed a slower response and lower improvement rates. In the present study, in multivariate analysis, CRP level over 100 mg/L was the only factor associated with non-improvement at day 7 after initiation of anti-*Leishmania* therapy for primary treatment of VL. High CRP has not been consistently suggested as a worse prognosis factor in previous studies; in a meta-analysis from East Africa [43] and a historical cohort from Brazil [44], prognostic factors for mortality among patients with VL included jaundice, edema, bleeding, splenomegaly, older age and *Leishmania*–HIV coinfection. However, findings in these populations may not be translatable to the Mediterranean context, considering baseline differences in sociodemographic aspects such as nutritional status and access to healthcare.

Secondary prophylaxis is common practice and endorsed by regional guidelines for PLWH [45]; for ISA, there is no consensus on indication, drug, frequency, and dosing and in the present study it was infrequently implemented; rate of relapse was similar between groups and in PLWH with or without prophylaxis. However, this possibly reflects the fact that in many cases prophylaxis could not be sustained until immunological recovery due to side effects, non-compliance, or dropout of patients. Even so, a longer time to relapse was documented in PLWH (compared to ISA) and especially in those on prophylaxis.

In most cases, treatment of relapses consisted of the use of the same or higher total dose of LAmB, but other regimens were used in selected cases. The results of the present study seem to suggest that improvement at 7 days could be higher with combination therapy, but a larger cohort would be needed. Randomized controlled trials could help understand if combination therapy is associated with better outcomes and whether there is any impact on subsequent relapse.

Although notification of VL cases increased compared to the previous period (49.7 vs. 38.6%) [12], approximately half of cases are still not reported, especially in the Norte and the AML regions, which could hamper public health efforts to control leishmaniasis in these regions. Incomplete and inconsistent reporting of VL increases the risk of bias in official data. Further studies should investigate causes for non-reporting, to better define strategies to tackle this gap in information.

Finally, this study presents some limitations, beginning with the fact that in some hospitals information was not collected due to lack of collaboration or due to absence of patient consent. In addition, coding of diagnosis for inpatients was not uniformly performed and digitalized in every hospital for the whole duration of the study period, and coding for outpatients was irregularly performed in hospitals, so cases were screened via laboratory results, whenever feasible. Some hospitals required internal personnel to access information, so in some cases interpretation of variables could be different, despite using the same database.

## Conclusions

Although globally in Portugal the incidence of VL decreased compared to the previous 10 years, the disease remains an individual, public and One Health problem and a marker of neglect. Rising incidence in the more recent years could be related to climate change, increased mobility and/or increase in susceptible groups. These factors could also favor a future geographic expansion of endemic *L. infantum* and the introduction of new *Leishmania* species.

Leishmaniasis presents a continuing threat in Portugal to PLWH and children and an increasing threat to other immunosuppressed groups. Disease in the latter poses specific problems in relation to diagnosis and treatment as a consequence of different clinical presentation, worse outcome, and general lack of scientific knowledge. Multicentric research efforts could provide evidence to optimize treatment strategies for these patients in the European context, especially concerning the use of secondary prophylaxis and treatment of relapses. Programs to control leishmaniasis should focus not only on reducing underreporting, but also on raising awareness for the disease among healthcare practitioners and providing tools for earlier diagnosis.

Systematically combining clinical and national surveillance data could allow a more detailed assessment of the epidemiologic situation and an evaluation of the progress in clinical practice, uncovering gaps that need to be addressed in the near future. In order to improve the overall outcome for leishmaniasis patients, human data should also be integrated with data from vectors and mammal hosts, to produce holistic strategies to control the disease in several parts of the life cycle, following a One Health approach.

### Supplementary Information

Additional file 1: Supplementary Figure 1. Maps showing the location of Mainland Portugal in Western Europe and the territorial division in NUTS (Nomenclature of Territorial Units for Statistics) 2 and NUTS3 regions.

Additional file 2: Supplementary Figure 2. Location ( a ) and microbiological agents ( b ) of coinfection/superinfection in primary visceral leishmaniasis episodes diagnosed between 2010 and 2020 ( n =194).

Additional file 3: Supplementary Table 1. List of NUTS (Nomenclature of Territorial Units for Statistics) 2 and NUTS3 regions in Mainland Portugal and sociodemographic characteristics.

Additional file 4: Supplementary Table 2. Definitions, classifications or categories used for data collection and presentation in this study.

## Data Availability

The datasets generated and analyzed during the current study are not publicly available due to confidentiality commitment with the health institutions and the participants.

## Abbreviations

AIDS: Acquired immunodeficiency syndrome
AML: Lisbon Metropolitan Area
a*OR*: Adjusted odds ratio
*CI*: Confidence interval
CL: Cutaneous leishmaniasis
CRP: C-reactive protein
CST: Pearson Chi-Square test
CU5: Children 5 years of age or younger
DGS: Directorate-General for Health
DNA: Desoxyribonucleic acid
ESCO: European Skills, Competences, Qualifications and Occupations
FET: Fisher’s exact test
HIV: Human immunodeficiency virus
HLH: Hemophagocytic lymphohistiocytosis
ICD: International Classification of Diseases
*IQI*: Interquartile interval
ISA: non-HIV infected immunosuppressed adults
KWT: Kruskal-Wallis test
LAmB: Liposomal amphotericin B
MWT: Mann-Whitney U test
NHS: National Health Service
NISA: Non-immunosuppressed adults and children over 5 years old
NUTS: Nomenclature of Territorial Units for Statistics
*OR*: Odds ratio
PCR: Polymerase chain reaction
PLWH: People living with HIV
SINAVE: National Epidemiologic Surveillance System
VL: Visceral leishmaniasis
WHO: World Health Organization
