# Supplementary material for: Epidemiological and clinical trends of visceral leishmaniasis in Portugal: retrospective analysis of cases diagnosed in public hospitals between 2010 and 2020
Source: Infect Dis Poverty. 2024 Jun 1;13:41. doi: 10.1186/s40249-024-01204-5 (PMC11143621; doi:10.1186/s40249-024-01204-5)
Supplement: Supplementary file 1 — Additional file 1: Supplementary Figure 1. Maps showing the location of Mainland Portugal in Western Europe and the territorial division in NUTS (Nomenclature of Territorial Units for Statistics) 2 and NUTS3 regions. [file 40249_2024_1204_MOESM1_ESM.docx]

**Supplementary figure 1**

Maps showing the location of Mainland Portugal in Western Europe (left) and the territorial division in NUTS (Nomenclature of Territorial Units for Statistics) 2 (middle) and NUTS3 (right) regions.

(NUTS – Nomenclature of Territorial Units for Statistics)


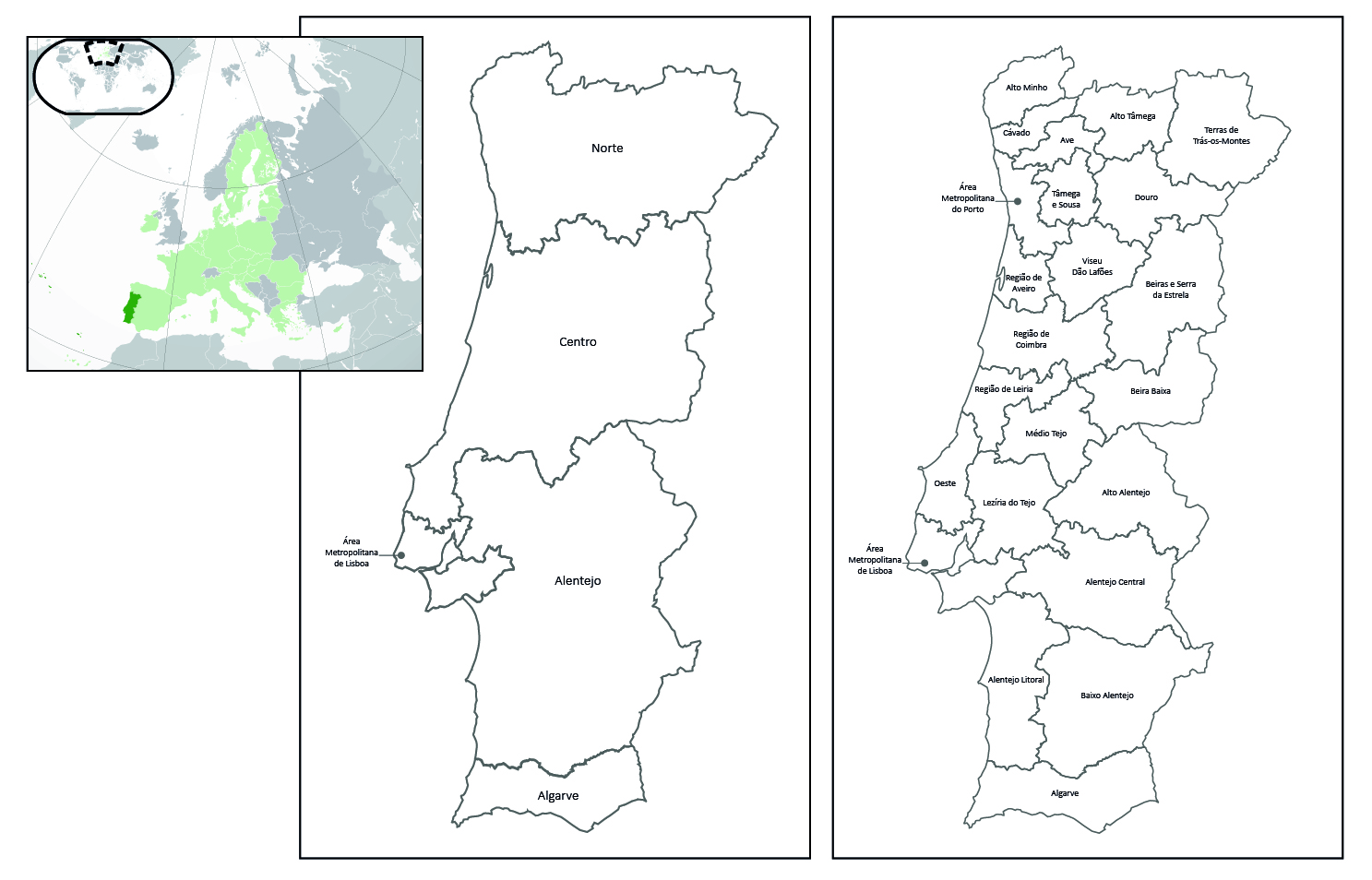


NUTS2

NUTS3
