# Supplementary figures and images for: Epidemiological and clinical trends of visceral leishmaniasis in Portugal: retrospective analysis of cases diagnosed in public hospitals between 2010 and 2020
Source: Infect Dis Poverty. 2024 Jun 1;13:41. doi: 10.1186/s40249-024-01204-5 (PMC11143621; doi:10.1186/s40249-024-01204-5)

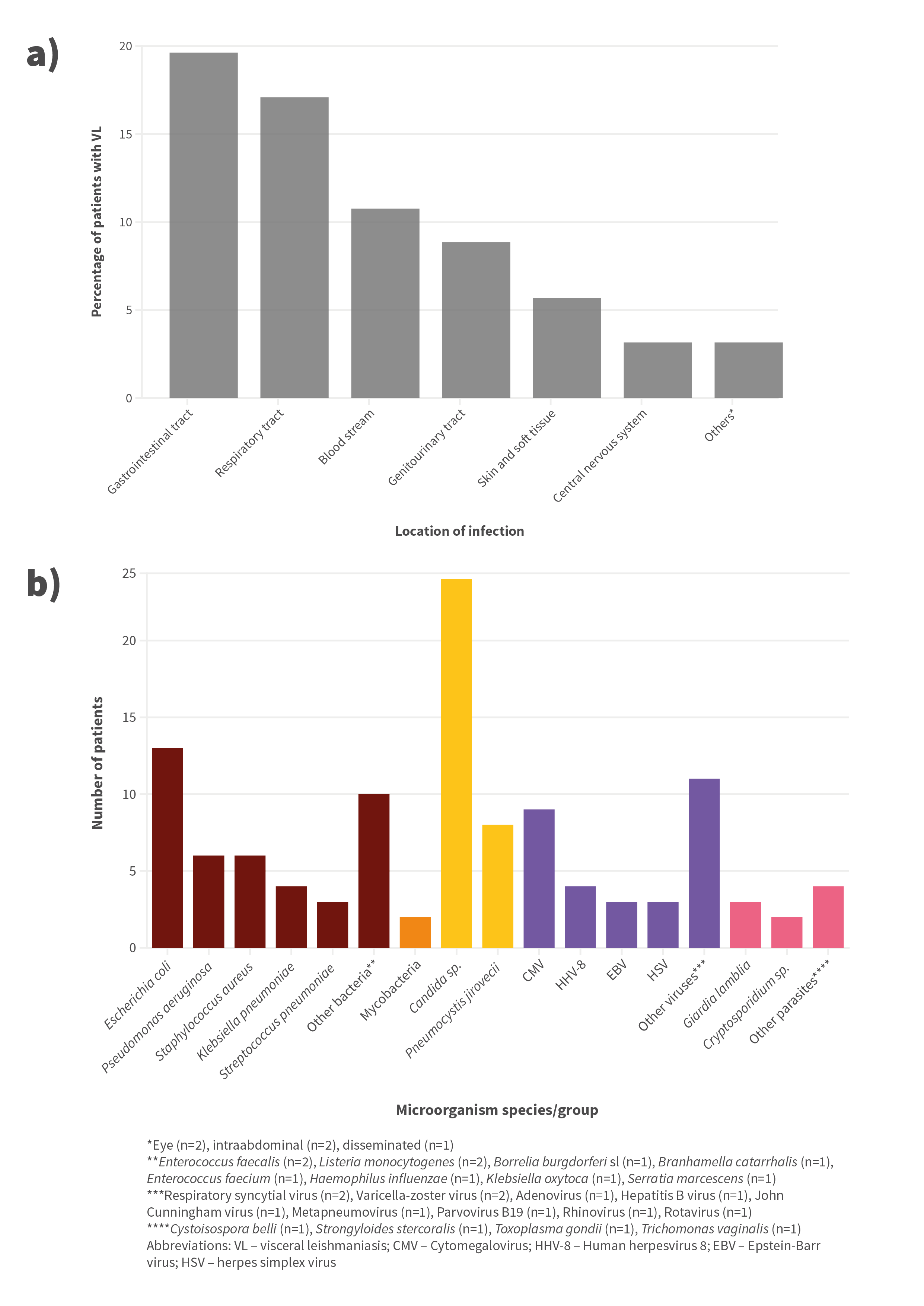

Supplement: Supplementary file 2 — Additional file 2: Supplementary Figure 2. Location ( a ) and microbiological agents ( b ) of coinfection/superinfection in primary visceral leishmaniasis episodes diagnosed between 2010 and 2020 ( n =194). [file 40249_2024_1204_MOESM2_ESM.png]
