# Supplementary material for: Epidemiological and clinical trends of visceral leishmaniasis in Portugal: retrospective analysis of cases diagnosed in public hospitals between 2010 and 2020
Source: Infect Dis Poverty. 2024 Jun 1;13:41. doi: 10.1186/s40249-024-01204-5 (PMC11143621; doi:10.1186/s40249-024-01204-5)
Supplement: Supplementary file 3 — Additional file 3: Supplementary Table 1. List of NUTS (Nomenclature of Territorial Units for Statistics) 2 and NUTS3 regions in Mainland Portugal and sociodemographic characteristics. [file 40249_2024_1204_MOESM3_ESM.docx]

**Supplementary table 1**

List of NUTS2 and NUTS3 regions in Mainland Portugal and sociodemographic characteristics.

| **Code** | **NUTS1** | **Code** | **NUTS2** | **Code** | **NUTS3** | **Population density*** | **Aging index*** |
| --- | --- | --- | --- | --- | --- | --- | --- |
| PT1 | Continente | PT11 | Norte | PT111 | Alto Minho | 104.2 | 251.9 |
|  |  |  |  | PT112 | Cávado | 334.4 | 146.5 |
|  |  |  |  | PT119 | Ave | 288.3 | 167.3 |
|  |  |  |  | PT11A | Área Metropolitana do Porto | 850.6 | 174.7 |
|  |  |  |  | PT11B | Alto Tâmega | 28.8 | 383.9 |
|  |  |  |  | PT11C | Tâmega e Sousa | 223.1 | 149.5 |
|  |  |  |  | PT11D | Douro | 45.6 | 274.4 |
|  |  |  |  | PT11E | [Terras de Trás-os-Montes](https://en.wikipedia.org/wiki/Terras_de_Tr%C3%A1s-os-Montes) | 19.4 | 359.0 |
|  |  | PT15 | Algarve | PT150 | Algarve | 93.5 | 176.7 |
|  |  | PT16 | Centro | PT16B | Oeste | 163.7 | 185.5 |
|  |  |  |  | PT16D | Região de Aveiro | 217.0 | 185.6 |
|  |  |  |  | PT16E | Região de Coimbra | 100.8 | 243.9 |
|  |  |  |  | PT16F | Região de Leiria | 117.1 | 201.8 |
|  |  |  |  | PT16G | Viseu Dão-Lafões | 78.1 | 246.3 |
|  |  |  |  | PT16H | Beira Baixa | 18.9 | 328.1 |
|  |  |  |  | PT16I | Médio Tejo | 78.6 | 249.0 |
|  |  |  |  | PT16J | Beiras e Serra da Estrela | 33.4 | 337.8 |
|  |  | PT17 | Área Metropolitana de Lisboa | PT170 | Área Metropolitana de Lisboa | 956.4 | 152.3 |
|  |  | PT18 | Alentejo | PT181 | Alentejo Litoral | 18.2 | 223.4 |
|  |  |  |  | PT184 | Baixo Alentejo | 13.4 | 217.9 |
|  |  |  |  | PT185 | Lezíria do Tejo | 55.2 | 199.6 |
|  |  |  |  | PT186 | Alto Alentejo | 17.2 | 253.5 |
|  |  |  |  | PT187 | Alentejo Central | 20.6 | 224.0 |

*According to data from the 2021 National Census (Instituto Nacional de Estatística. Censos 2021 Resultados Definitivos - Portugal. Lisboa; 2022)
