# Supplementary material for: Epidemiological and clinical trends of visceral leishmaniasis in Portugal: retrospective analysis of cases diagnosed in public hospitals between 2010 and 2020
Source: Infect Dis Poverty. 2024 Jun 1;13:41. doi: 10.1186/s40249-024-01204-5 (PMC11143621; doi:10.1186/s40249-024-01204-5)
Supplement: Supplementary file 4 — Additional file 4: Supplementary Table 2. Definitions, classifications or categories used for data collection and presentation in this study. [file 40249_2024_1204_MOESM4_ESM.docx]

**Supplementary table 2**

Classifications, categories and definitions used for data collection and presentation in this study.

| **Concept/variable** | **Definition, classifications or categories** |
| --- | --- |
| Migrant | Person born abroad |
| Prior travel to or residence in *Leishmania* *donovani* complex endemic areas | Defined as a stay of any duration in: countries in Europe where disease has been reported in most or all regions - Albania, Bulgaria, Cyprus, Greece, Italy, Malta and Spain (1); countries outside Europe where over 200 cases of VL have been reported to the WHO in 2021 (2) - Brazil, Eritrea, Ethiopia, India, Kenya, Nepal, Somalia, South Sudan, Sudan and Yemen (3). |
| Autochthonous case | No species other than *L. infantum* was identified and: a) there was no lifetime history of travel or residence abroad in *Leishmania* endemic regions (as defined above); b) there was a history of travel or residence in *Leishmania* endemic region(s), but it occurred more than 12 months before the beginning of symptoms and there was no change in immune status since the stay abroad; c) or there was no information regarding travel history |
| Imported case | Case not meeting the criteria listed above |
| Time to presentation | The amount of time elapsed since the beginning of signs/symptoms or laboratory abnormalities related to VL and first visit to healthcare providers/institutions. |
| Time to diagnosis | The amount of time elapsed since presentation to healthcare providers and confirmation of the diagnosis of VL, considering the date when results were made available (not when samples were obtained). |
| Time to treatment | The amount of time elapsed since confirmation of diagnosis and start of *Leishmania* directed therapy |
| Fever | Defined as body temperature above 38ºC (4) (or assumed when written in the records). |
| Hepatosplenomegaly | Considered only when stated in the report of a radiological exam. |
| Lymphadenopathy | Assumed when reported on a radiological exam (internal/superficial lymph nodes) or described in a medical physical examination (superficial). |
| Hemophagocytic lymphohistiocytosis (HLH) | Considered when mentioned in clinical records or if HLH-2004 criteria were fulfilled (5). |
| Immunosuppressed patient | For purposes of this study, if one or more of the following conditions were present: HIV infection with a CD4 cell count <500/µL; any primary immunodeficiency; active solid or hematologic malignancy; prior solid organ or bone marrow transplantation; current treatment with immunosuppressive/immunomodulatory drugs (as listed in (6)). |
| Rural or non-rural parish | Classification followed the Portuguese Rural Development Program 2014–2020 (7). |
| Professional occupations | Classified using the European Skills, Competences, Qualifications and Occupations (ESCO) classification, developed by The European Commission since 2010 (8) |
| NUTS regions, municipalities, parishes, and unions of parishes | Defined according to the organizational definition published in 2013 and implemented in 2015.  (The order of presentation of NUTS2 and 3 regions in tables follows the numerical and/or alphabetical order of their respective official codes) |

References

1. ECDC. Surveillance, prevention and control of leishmaniases in the European Union and its neighbouring countries. Stockholm; 2022.
2. WHO. THE GLOBAL HEALTH OBSERVATORY. 2023 [cited 2023 Mar 24]. Number of cases of visceral leishmaniasis reported. Available from: [https://www.who.int/data/gho/data/indicators/indicator-details/GHO/number-of-cases-of-visceral-leishmaniasis-reported. Accessed 3 Mar 2024](https://www.who.int/data/gho/data/indicators/indicator-details/GHO/number-of-cases-of-visceral-leishmaniasis-reported.%20Accessed%203%20Mar%202024).
3. World Health Organisation. Status of endemicity of cutaneous leishmaniasis: 2022 [Internet]. 2023 [cited 2023 Oct 8]. Available from: <https://apps.who.int/neglected_diseases/ntddata/leishmaniasis/leishmaniasis.html>. Accessed 3 Mar 2024.
4. Garner JS, Jarvis WR, Emori TG, Horan TC, Hughes JM. CDC definitions for nosocomial infections, 1988. Am J Infect Control. 1988 Jun;16(3):128–40.
5. Henter J, Horne A, Aricó M, Egeler RM, Filipovich AH, Imashuku S, et al. HLH‐2004: Diagnostic and therapeutic guidelines for hemophagocytic lymphohistiocytosis. Pediatr Blood Cancer. 2007 Feb 25;48(2):124–31.
6. Nemhauser J. Travelers with Additional Considerations. In: CDC Yellow Book 2024. Oxford University PressNew York; 2023. p. 139–80.
7. Programa de desenvolvimento rural 2014-2020 [Internet]. Freguesias Rurais PDR2020 (nova Divisão Administrativa freguesias 2013). Available from: <https://www.gpp.pt/images/Estatisticas_e_analises/Estatisticas/associadasmedidasapoio/Territorios_Rurais.pdf>. Accessed 3 Mar 2024. (in Portuguese)
8. ESCO. Classification of Occupations [Internet]. Available from: <https://esco.ec.europa.eu/en/classification/occupation_main>. Accessed 3 Mar 2024.
